# Seroprevalence of *Bordetella pertussis* toxin antibodies in children and adolescents in Tunis, Tunisia

**DOI:** 10.1017/S0950268819000840

**Published:** 2019-05-17

**Authors:** I. Ben Fraj, M. Zghal, M. Hsairi, A. Kechrid, H. Smaoui

**Affiliations:** 1 University of Tunis El Manar, Children's Hospital of Tunis, Laboratory of Microbiology, UR12ES01, Tunis, Tunisia; 2 Department of Epidemiology, Salah Azaiz Institute of Cancer, Tunis, Tunisia

**Keywords:** Adolescents, children, pertussis, seroprevalence, Tunisia

## Abstract

Pertussis remains a public health concern in most countries. This cross-sectional study aims to investigate the distribution of pertussis toxin antibodies (anti-PT IgG) in Tunisian children and adolescents aged 3–18 years, to define optimal age for booster vaccination. Anti-PT IgG concentrations of enrolled participants were measured using commercial enzyme-linked immunosorbent assay. Concentrations were classified as: indicative of current/recent infection if ⩾100 IU/ml, indicative of recent exposure to *Bordetella pertussis* within the last year if 40–100 IU/ml and less likely revealing a recent exposure to *B. pertussis* if <40 IU/ml. Between March and June 2018, a total of 304 participants (mean age: 9.3 years) were included in this study. Overall, 12.8% (95% confidence interval (CI) 9.1%–16.6%) were seropositive (IgG levels ⩾40 IU/ml). Among them, 14.7% (95% CI 2.3%–23.3%) had levels indicative of a current/recent infection. The multivariate Poisson regression analysis suggested associations between female gender, as well as age group 13–18 years and 3–5 years and higher anti-PT IgG concentrations. Our results are consistent with the notion that vaccine-induced immunity decline, as well as circulation of pertussis among school children and adolescents enables them to be reservoirs of infection and disease transmission to vulnerable infants. Booster dose of acellular pertussis vaccine for school entrants is therefore recommended.

## Introduction

Pertussis, mainly caused by the bacterium *Bordetella pertussis*, is a highly contagious infectious disease [1]. Despite high-vaccination rates, pertussis remains a public health concern in several countries. New-borns and infants, not or partially vaccinated, are mostly affected [2]. They have the highest risk of eventual malignant pertussis manifested by severe complications including seizures, pneumonia, encephalopathy and a marked leucocytosis [3, 4].

An increase in pertussis incidence among old children and adolescents in some countries has been well-described [5]. In fact, these age categories, especially family members, seem to be a potential source of pertussis transmission to young infants [2, 6]. Contributing factors of this increase could be waning of vaccine-induced immunity, pathogen adaptation and changes in disease susceptibility [5, 7].

In Tunisia, infants are immunised with whole cell pertussis (wP) as a pentavalent combined vaccine (DTwP-HepB-Hib, Pentavac^®^, Serum Institute of India) at the age of 2, 3 and 6 months according to the National Immunization Program (NIP). A booster dose including diphtheria, tetanus and wP (DTwP) is administered at the age of 18 months [8]. Despite high-national vaccination coverage (98%), circulation of *B. pertussis* in Tunisian infants has been reported [9, 10] and three epidemics have been observed respectively in 2009, 2014 [10] and 2018 (unpublished observations). Moreover, circulation of pertussis among healthcare workers has been recently reported in a seroepidemiological study [11]. However, little is known about pertussis seroprevalence among children and adolescents, who are a potential reservoir of the disease.

Pertussis immunity, whether induced by vaccination or natural infection, is not lifelong [7]. Therefore, seroepidemiology is a powerful tool used to monitor effectiveness of vaccination programmes and identify age groups contributing to disease transmission [12]. In fact, determination of pertussis seroprevalence in a given population helps assess the immunity level and identify the target population for booster vaccination to reduce individual morbidity [12]. Moreover, small serosurveys in blood donors is a simple method for estimating a recent exposure to *B. pertussis* [13]. Unlike Tunisia, various countries have introduced booster doses of acellular pertussis vaccine (aP) for older children, adolescents and/or adults [14, 15].

This study aims to determine prevalence of immunoglobulin G (IgG) antibodies to pertussis toxin (PT) amongst a cohort of pre-school, school children and adolescents to evaluate the distribution of anti-PT IgG in different age groups and to assess age of decrease of vaccine-acquired immunity.

## Materials and methods

### Study design

This cross-sectional study was carried out from March 2018 to June 2018. Individuals aged 3–18 years, not having current respiratory infection and visiting the Children's Hospital of Tunis for check-up, were enrolled. All participants had received whole-cell pertussis primo-vaccination according to NIP. Information about age, DTwP booster vaccine history, sibling and history of long-lasting cough was obtained using a questionnaire.

### Laboratory methods

Blood samples were collected via venepuncture and transported immediately to the Laboratory of Microbiology at the Children's Hospital of Tunis. After centrifugation, sera were extracted and stored at −20 °C until analysis. Measurement of anti-PT IgG concentrations was performed by using a commercial enzyme-linked immunosorbent assay (ELISA) kit (Euroimmun, Lübeck, Germany) according to the manufacturer's protocol. Concentrations were reported in International Units/ml (IU/ml). Results were interpreted following the manufacturer's instructions and as previously described [16]. Anti-PT IgG levels ⩾100 IU/ml were indicative of recent or active *B. pertussis* infection if the participant did not receive pertussis booster vaccine within the last 12 months. Concentrations between 40 and 100 IU/ml were considered to indicate a recent contact with *B. pertussis* within the last year, while anti-PT IgG levels <40 IU/ml were less likely revealing a recent exposure to *B. pertussis*. In fact, the cut-offs used were intended for diagnostic purposes, and levels below 40 IU/ml didn't mean seronegativity in an epidemiological sense, however they made a recent exposure to *B. pertussis* less likely.

### Statistical analysis

Data were analysed using SPSS Software version 25 (SPSS Inc., Chicago, USA), with *P* < 0.05 considered to be significant. Subjects were stratified into three groups according to their ages. Values below the level of detection (5 IU/ml) were given a value of 2.5 IU/ml, whereas concentrations above the upper limit (200 IU/ml) were given a value of 175 IU/ml. Association of IgG anti-PT levels with gender, DTwP booster history, sibling, history of long-lasting cough and age groups was determined using the Pearson's chi-squared test (*χ*^2^). We used logistic as well as Poisson regression analysis to determine whether there are risk factors for children and adolescents that are associated with an increased chance on acquiring pertussis infection. Variables were expressed as odds ratios (OR) with their 95% confidence interval (CI).

## Results

**Fig. 1.**
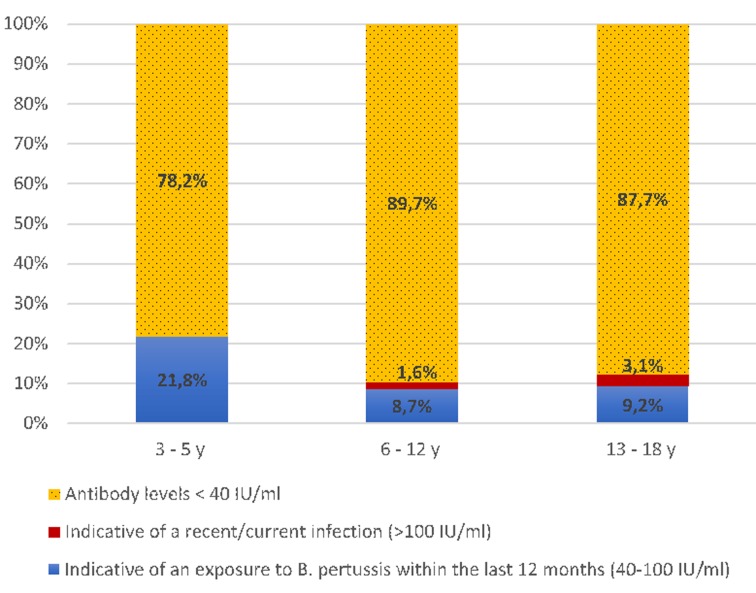
Frequency of anti-PT IgG levels in different age groups.

A total of 304 participants aged 3–18 years were enrolled in the current study. Mean age was 9.3 years and 51.3% were female. Population was divided into three age groups: pre-school children: 3–5 years (*n* = 55), school children: 6–12 years (*n* = 184) and high-school children: 13–18 years (*n* = 65). Distribution of ELISA results according to age categories is shown in Figure 1.

Overall, 39 (12.8%; 95% CI 9.1%–16.6%) subjects had antibody concentrations of ⩾40 IU/ml. Among them, 34 (11.2%) had ELISA results indicative of an infection/vaccination within the last 12 months, whereas five (1.6%) had ELISA results compatible with a recent or current infection (⩾100 IU/ml). Distribution of serology results according to age categories was not statistically significant (*P* > 0.05).

Using univariate and multivariate logistic regression analysis (Table 1), no significant association has been observed between seropositivity and demographic factors including gender, age category, sibling under 5 years and history of long-lasting cough (*P* > 0.05).

**Table 1.** Association between demographic factors and seropositivity: univariate and multivariate analysis

Variables	Univariate analysis			Multivariate analysis		
	OR	95% CI of OR	*P* value	OR	95% CI of OR	*P* value
Gender						
Male	1	–	–	1	–	–
Female	0.888	0.453–1.739	0.7282	0.876	0.439–1.750	0.7077
Age category						
13–18 years	1	–	–	1	–	–
3–5 years	1.988	0.748–5.289	0.1685	2.314	0.828–6.467	0.1096
6–12 years	0.820	0.341–1.977	0.6591	0.870	0.356–2.124	0.7593
History of prolonged cough	1.059	0.518–2.162	0.8753	0.975	0.465–2.043	0.9457
Existence of sibling ⩽5 years	0.794	0.348–1.814	0.5843	0.692	0.288–1.663	0.4101

**Fig. 2.**
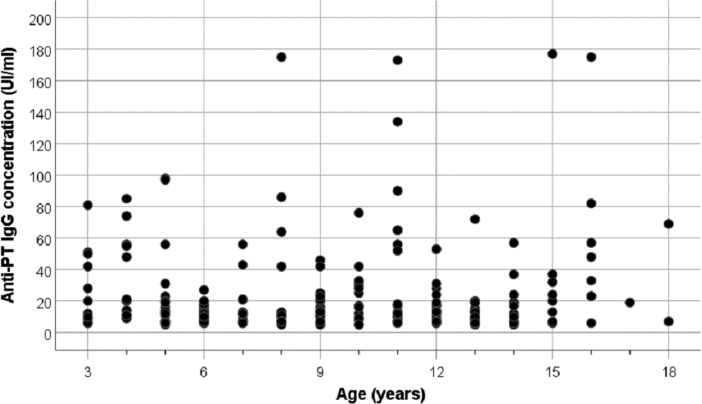
Distribution of anti-PT IgG levels according to age.

The highest IgG titres (⩾40 IU/ml) were detected among individuals aged 8–18 years (Fig. 2). Table 2 shows arithmetic and geometric mean anti-PT IgG titres in female, male and various age categories. No significant differences between groups were observed (*P* > 0.1). Overall, 107 (35.2%) cases were found to have undetectable anti-PT IgG antibodies (<5 IU/ml). The proportions were significantly highest in participants aged 7, 8 and 12 years (*P* = 0.001), confirming the hypothesis of rapid loss of immunity over time.

**Table 2.** Arithmetic and geometric anti-PT IgG titre means according to gender and age groups

Variable	Number	Arithmetic titres (IU/ml)			Geometric titres (IU/ml)		
		Mean	Std	Median	Mean	Std	Median
Gender							
Male	148	18.1	27.9	9.0	9.0	3.3	8.2
Female	156	17.1	26.6	7.0	8.2	3.3	6.7
*P* value	0.75				0.54		
Age groups							
3–5 years	55	21.8	26.2	11.0	11.0	3.3	11.0
6–12 years	184	15.4	24.9	7.0	7.4	3.0	6.7
13–18 years	65	20.3	33.4	7.0	9.0	3.3	6.7
*P* value	0.21				0.15		
Total	304	17.6	27.2	8.0	8.2	3.3	8.2

Given the fact that a pertussis outbreak has started during the study period, we performed Poisson regression model to assess the impact in anti-PT IgG levels according to time, reflecting exposure to *B. pertussis*, to demographic factors (including age groups, gender, prolonged cough and sibling ⩽5 year) and to interaction time × age groups (Table 3). Our findings suggested that age groups 3–5 years, 13–18 years and female gender were significantly positively associated with increased anti-PT IgG levels. While the interaction delay×age group 13–18 years was negatively associated with anti-PT IgG levels.

**Table 3.** Factors associated with changes in anti-PT IgG titres during the outbreak – Poisson regression model^a^

Factors	Coefficient (95% CI)	*P* value
Female	0.0893 (0.0424–0.0063)	<0.0001
Age category 3–5 years^b^	0.3859 (0.2807–0.4912)	<0.0001
Age category 13–18 years	0.2092 (0.1084–0.3099)	<0.0001
Delay^c^	0.0027 (−0.0006 to 0.0061)	0.1088
Prolonged cough	0.0718 (−0.0155 to 0.1591)	0.1069
Sibling ⩽5 years	0.0203 (−0.0731 to 0.1137)	0.6703

a Patients investigated before the starting of outbreak were excluded.

b Age group 6–12 years and masculine gender have been considered as reference categories for comparison.

c Time between patients' investigation and outbreak starting.

## Discussion

As in other countries, possible explanations of pertussis cases increase in Tunisia include: improved diagnosis testing, better awareness of medical community, increased reporting of pertussis cases and waning of vaccine-induced immunity [7, 9, 17]. In fact, immunity induced by pertussis infection or vaccination is not lifelong [7]. In our study, we focused on the assessment of anti-PT IgG levels, specific marker of *B. pertussis* infection/vaccination with no reported cross-reactivity [16], in participants aged 3–18 years. To our knowledge, the current study is the first to provide information about seroprevalence of pertussis among Tunisian children and adolescents.

Among the study population, 87.2% (95% CI 83.4%–90.9%) had anti-PT concentrations <40 IU/ml. The highest rate (89.7%; 95% CI 85.3%–94.1%) was observed among school children aged 6–12 years. This suggests rapid decrease of vaccine-acquired immunity after the last DTwP booster dose received at 18 months. This is consistent with the study of Schwartz *et al*., who reported that pertussis vaccine effectiveness declines after 4 years and there is little to no protection beyond 7 years from last vaccination [18].

Demographic characteristics including age, gender, sibling under 5 years and history of long-lasting cough were not significantly associated with serology rates (*P* > 0.05). Varied results have been described elsewhere [13, 19, 20].

Our findings revealed that 12.8% (95% CI 9.1%–16.6%) of study population were seropositive (IgG levels ⩾40 IU/ml). The most increased seropositivity rate (21.8%; 95% CI 10.9%–32.7%) was observed among children aged 3–5 years. This is likely due to the persisting vaccine-acquired immunity, that still be observed within 3 years from the last vaccination dose [18, 21]. Increased anti-PT IgG levels have been observed in countries using the acellular vaccine, including the Netherlands [22] and Germany [13]. Moreover, it has been reported that DTwP primed-children are better protected against pertussis than DTaP primed adolescents [23].

Our results confirmed an overall significant increase in titres according to female and age categories 3–5 years and 13–18 years. However, associations of following variables: delay, sibling ⩽5 years and prolonged cough were not significant. In fact, data from seroprevalence studies suggest that re-exposure to *B. pertussis* can boost antibody titres, without developing clinical symptoms or causing a transmissible infection. Therefore, prolonged cough was not a significantly associate variable [24].

Besides, the results of age stratified Poisson regression analysis revealed that the coefficient for delay × age group 3–5 years is positive, suggesting an increase in titres as time progressed (Table 4). However, the coefficient for delay × age group 13–18 years was negative meaning a temporal decline in titres for participants aged 13–18 years, suggesting that those adolescents played a disproportionate role in propagating the epidemic. The latter would be consistent with previous studies [25, 26]. Besides, various models studying the immune response to *B. pertussis* infection provided evidence that secondary exposure is less likely to boost immunity than primary exposure, given equal levels of contact with *B. pertussis*. Moreover, immunity to pertussis wanes before re-exposure occurs, because of low pathogen circulation [24] (Table 4).

**Table 4.** Stratified analysis of changes in anti-PT IgG titres during the outbreak and time between patients' investigation and outbreak starting – Poisson regression model

	Factors	Coefficient (95% CI)	*p* value
Delay	Age group 3–5 years	0.0168 (0.0108–0.0227)	<0.0001
	Age group 6–12 years	0.0026 (−0.0024 to 0.0076)	0.3149
	Age group 13–18 years	−0.0143 (−0.0218 to −0.0069)	0.0002

Delay = time between patients' investigation and outbreak starting.

Children and adolescents represent a potential source of infection of infants and toddlers around them. Given the fact that Tunisia is a high vaccine coverage country [27], pertussis occurrence among this age category is likely due to waning immunity after both vaccination and infection [12]. Therefore, increased awareness of medical community is required to involve the differential diagnosis of chronic cough caused by pertussis disease and treat it with macrolides. Besides, introducing a booster dose for Tunisian school children would be efficient to strengthen their immunity and protect new-borns and infants too young to be fully vaccinated.

Waning of vaccine-acquired immunity could be rectifiable by booster vaccination with less reactogenic aP-containing vaccines [28]. The Global Pertussis Initiative recommends booster vaccination of persons at risk, including school entrants and adolescents based on available logistics, resources and access to aP vaccines [29]. In Tunisia, booster vaccination is not logistically feasible in the absence of TdaP (tetanus-diphtheriae-acellular pertussis) vaccine. Therefore, it would be suitable to consider introducing it in our country, where pertussis is occurring in infants and children in epidemiologic waves [10].

Our study presents preliminary results about pertussis seroprevalence in a cohort of Tunisian children and adolescents during the second quarter of 2018. Nevertheless, it has some limitations. Sampling was based on population attending a university paediatric hospital. Therefore, the results may not represent the whole population. However, they are consistent with seroepidemiological studies performed elsewhere. Moreover, enrolled participants were asymptomatic and had no current respiratory infectious disease to avoid bias. Another limitation was the small study population. Therefore, further serosurveys with larger populations are required to better estimate the optimal age for booster vaccination and adapt the national vaccination schedule.

## Conclusions

The evidence of pertussis infection in Tunisian school children and adolescents confirms the rapid decline of wP vaccine-acquired immunity and highlights the importance of increased awareness among medical community about this disease. Given the fact that high-school children aged 13–18 years had a higher attack rate during the pertussis epidemic, a TdaP booster dose would be suitable to reinforce their immunity and protect young infants, for whom pertussis could be deadly.
